# Ad26.COV2.S COVID-19 Vaccine Safety And Immunogenicity in Adolescents 16–17 Years of Age

**DOI:** 10.1093/jpids/piae098

**Published:** 2024-09-28

**Authors:** Javier Ruiz-Guiñazú, Mathieu Le Gars, Vicky Cárdenas, Nathalie Vaissière, Jerald Sadoff, Carla Truyers, Jenny Hendriks, Gert Scheper, A Marit de Groot, Frank Struyf, Hanneke Schuitemaker, Macaya Douoguih

**Affiliations:** Janssen Research & Development, Beerse, Belgium; Janssen Vaccines & Prevention Leiden, The Netherlands; Janssen Research & Development, Spring House, Pennsylvania, USA; Janssen Research & Development, Beerse, Belgium; Janssen Vaccines & Prevention Leiden, The Netherlands; Janssen Research & Development, Beerse, Belgium; Janssen Vaccines & Prevention Leiden, The Netherlands; Janssen Vaccines & Prevention Leiden, The Netherlands; Janssen Vaccines & Prevention Leiden, The Netherlands; Janssen Research & Development, Beerse, Belgium; Janssen Vaccines & Prevention Leiden, The Netherlands; Janssen Vaccines & Prevention Leiden, The Netherlands

**Keywords:** Ad26.COV2.S, adolescents, COVID-19, infectious diseases, vaccine/immunization

## Abstract

2.5 × 10^10^ vp Ad26.COV2.S elicited robust SARS-CoV-2–specific antibody responses in adolescents through 6 months, with acceptable safety and reactogenicity profiles. Compared with adults immunized with 5 × 10^10^ vp Ad26.COV2.S, adolescents had higher antibody levels, despite being vaccinated with a lower dose.

Ad26.COV2.S is a recombinant, replication-incompetent, human adenovirus type 26 (Ad26)–vectored vaccine encoding a prefusion conformation–stabilized SARS-CoV-2 spike protein [1]. A randomized, placebo-controlled, phase 2a trial of healthy adults (NCT04535453) was amended to include a sentinel group of adolescents aged 16–17 years for evaluation of the short-term safety and immunogenicity of a single dose of 2.5 × 10^10^ vp Ad26.COV2.S. Findings in this adolescent cohort were compared to results in young adult (18–25 years) and adult (18–55 years; ≥65 years) participants enrolled in the same study.

Eligible participants aged 16–17 years were randomized 10:1 to receive 1 dose of 2.5 × 10^10^ vp Ad26.COV2.S or placebo (Supplementary Methods). The primary and secondary objectives for the adolescent cohort were to assess safety and humoral immune responses, respectively. The full analysis set included all participants with documented vaccination. The per-protocol immunogenicity (PPI) set included all participants with available immunogenicity data. An independent data monitoring committee determined if the immunogenicity and reactogenicity profiles observed post-vaccination in participants 16–17 years were acceptable (Supplementary Methods).

Forty-four adolescent participants were screened, of whom 33 were randomized to receive vaccine (*n* = 30) or placebo (*n* = 3) (Supplementary Results; Supplementary Table 1).

The most frequently reported solicited local adverse event (AE) was vaccination-site pain; most AEs were grade 1 or 2 in severity (for grading scales, see Supplementary Tables 2 and 3). Headache and fatigue were the most commonly reported solicited systemic AEs. No serious AEs were reported in adolescents. This study was not powered to detect rare but significant AEs associated with this vaccine, such as vaccine-induced immune thrombotic thrombocytopenia.

Local and systemic reactogenicity were observed in a higher proportion of adolescents compared with adults or young adults aged 18–25 years who were vaccinated with 5 × 10^10^ vp Ad26.COV2.S (Figure 1). Adolescents and younger adults both reported higher rates of solicited AEs than older adults. Unsolicited AEs are presented in Supplementary Table 4.

**Figure 1.**
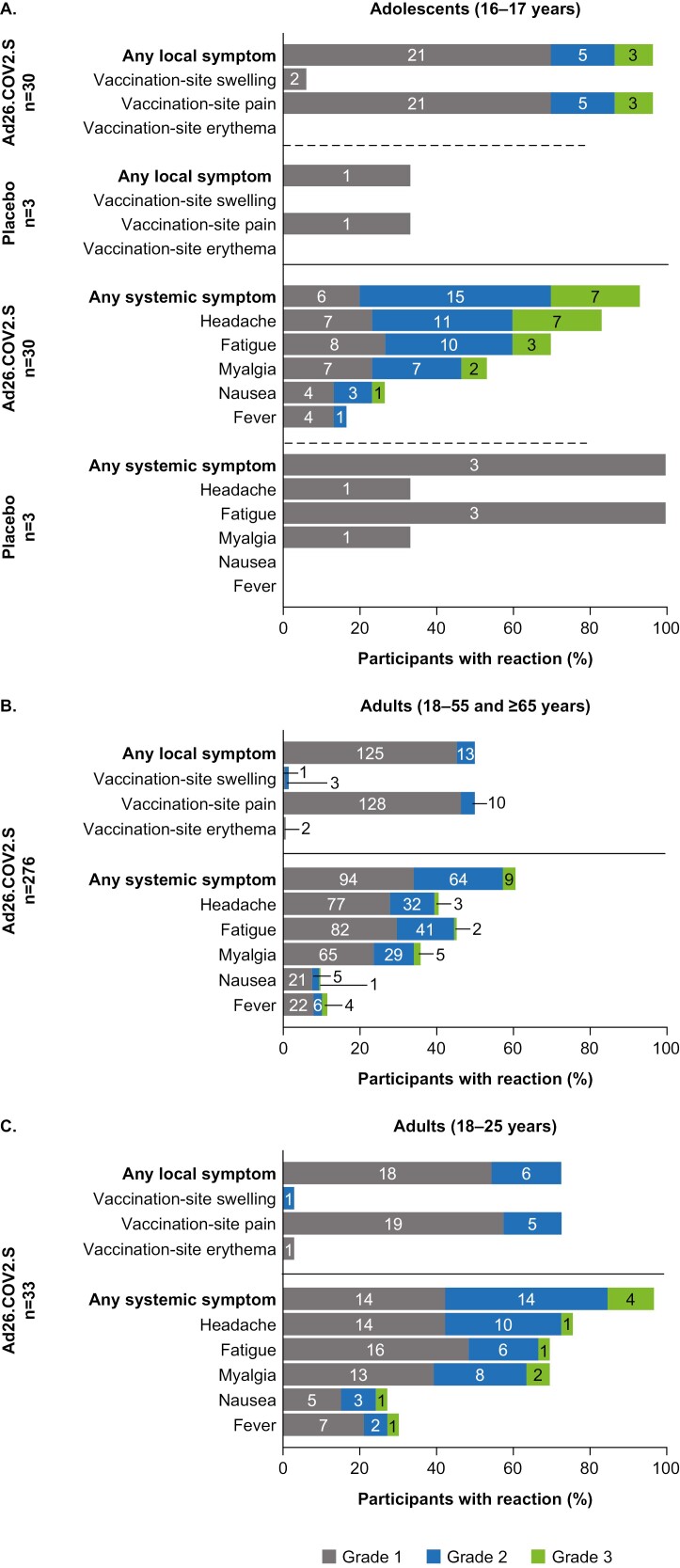
Solicited adverse events in (A) adolescent participants who received 2.5 × 10^10^ vp Ad26.COV2.S vaccine or placebo; (B) healthy adults aged 18–55 and ≥65 years who received 5 × 10^10^ vp Ad26.COV2.S vaccine; and (C) healthy adults aged 18–25 years who received 5 × 10^10^ vp Ad26.COV2.S vaccine. The number of participants reporting each grade is shown within each section of bars. Abbreviations: vp, viral particles.

Thirty baseline-seronegative participants (vaccine, *n* = 27; placebo, *n* = 3) were included in the PPI set. In the vaccine group, geometric mean concentrations (GMCs; EU/mL) of spike protein-specific binding antibodies, assessed by a spike protein-specific enzyme-linked immunosorbent assay (S-ELISA), were 682 (95% CI: 506–920; responder rate, 100%) at Day 29 and were maintained up to Day 169 (Supplementary Figure 1). Overall, GMCs of spike protein-binding antibodies in vaccinated adolescents tended to be higher than in adults (18–55 and >65 years) who received a single dose of 5 × 10^10^ vp.

Neutralizing antibodies were assessed by wild-type virus-neutralization assay. By Day 29, geometric mean titers (GMTs [95% CI]) were 305 (245–378) in vaccinated adolescents and remained stable through Day 169 (Supplementary Figure 2). GMTs in adolescents tended to be higher than in adults 18–55 and ≥65 years; however, from Day 29 through Day 169, neutralizing antibodies in adolescents trended lower than in young adults aged 18–25 years.

Previous phase 3 studies have demonstrated that primary and booster vaccinations of 5 × 10^10^ vp Ad26.COV2.S are immunogenic and efficacious against moderate-to-severe–critical COVID-19 disease in adults [2–4]. Here, we have shown that a lower dose level of 2.5 × 10^10^ vp in adolescents had an acceptable short-term safety profile and elicited robust humoral immune responses up to Day 169, supporting efficacy in individuals aged 16–17 years.

In this study, reactogenicity of a single dose of 2.5 × 10^10^ vp Ad26.COV2.S in adolescents tended to be higher than a single dose of 5 × 10^10^ vp in adults. Overall, the short-term safety and reactogenicity profile in adolescents was acceptable compared to adults aged 18–55 and ≥65 years who received 5 × 10^10^ vp Ad26.COV2.S [3]. The rate of solicited systemic grade 3 AEs (23.3%) in vaccinated adolescents may suggest the adenovirus vector, the spike protein, or the combination thereof is not as well tolerated in younger versus older vaccinees.

Compared to humoral immune responses in adults (18–55 and ≥65 years) observed after vaccination with 5 × 10^10^ vp Ad26.COV2.S, adolescents receiving half the adult dose had higher antibody levels (2- to 2.4-fold) and response rates through Day 169. This trend is similar to other studies that have reported higher antibody responses in adolescents versus younger adults receiving an equivalent dose of mRNA COVID-19 vaccines [5, 6]. Because binding and neutralizing antibody levels in this study were higher in magnitude at Day 169 for adolescents versus adults, vaccine responses may also be more durable in adolescents versus adults.

The main limitation of the data reported here is the small number of participants and narrow age range, contributing to a lack of diversity among the participants. Results should be interpreted with caution, considering the small sample size and a placebo arm that included only 3 participants. Importantly, although comparisons of immune responses to adults are informative, they require adequately powered noninferiority trials for confirmation.

## Supplementary Data

Supplementary materials are available at the *Journal of The Pediatric Infectious Diseases Society* online (http://jpids.oxfordjournals.org).

piae098_suppl_Supplementary_Materials

## Notes

***Financial support.*** This study is supported by Janssen Vaccines & Prevention BV in collaboration with the Biomedical Advanced Research and Development Authority, the National Institutes of Health, the Department of Defense, and the COVID-19 Prevention Network. This project has been funded in whole or in part with federal funds from the Office of the Assistant Secretary for Preparedness and Response, Biomedical Advanced Research and Development Authority, under Contract No. HHSO100201700018C.

***Potential conflicts of interest.*** J.R.-G. is a current employee of Johnson & Johnson and holds Johnson & Johnson stock or stock options, and is a former employee of GSK and holds GSK stock or stock options. M.L.G, J.S., H.S., and M.D. were employees of Janssen Vaccines & Prevention at the time of the study and may hold shares or restricted shares in Johnson & Johnson. V.C. and F.S. were employees of Janssen Research & Development at the time of the study and may hold shares or restricted shares in Johnson & Johnson; F.S. also holds shares from the GSK group of companies as part of past employee remuneration. N.V. was a consultant for Johnson & Johnson at the time of the study. C.T. is a current employee of Janssen Research & Development and J.H., G.S., and A.M.dG. are current employees of Janssen Vaccines & Prevention, part of the Janssen Pharmaceuticals family of companies of Johnson & Johnson, and may hold shares or restricted shares in Johnson & Johnson.

***Role of funder/sponsor***. The sponsor of the study was responsible for study design, study conduct, data collection, data analysis, and data interpretation, and authors employed by the sponsor contributed to the writing of the report and the decision to submit for publication.

***Clinical trial registration*.** ClinicalTrials.gov Identifier: NCT04535453.

***Data-sharing statement*.** The data-sharing policy of Janssen Pharmaceutical Companies of Johnson & Johnson is available at https://www.janssen.com/clinical-trials/transparency. As noted on this site, requests for access to the study data can be submitted through Yale Open Data Access Project site at http://yoda.yale.edu.
