# Supplementary material for: Ad26.COV2.S COVID-19 Vaccine Safety And Immunogenicity in Adolescents 16–17 Years of Age
Source: J Pediatric Infect Dis Soc. 2024 Sep 28;13(11):599–601. doi: 10.1093/jpids/piae098 (PMC11599148; doi:10.1093/jpids/piae098)

**Supplementary Material to: Ad26.COV2.S COVID-19 vaccine safety and immunogenicity in adolescents 16–17 years of age**

Javier Ruiz-Guiñazú, et al.

**Supplementary Methods.**

## Trial Design and Participants

The trial (COV2001; ClinicalTrials.gov Identifier: NCT04535453) was initiated on August 31, 2020, in Germany, The Netherlands, and Spain in healthy adults aged 18–55 and ≥65 years. The study was approved by local institutional review boards and/or ethics committees. An amendment to the study protocol to initiate enrollment of participants aged 16–17 years was approved by ethics committees in Spain and the United Kingdom and began on March 3, 2021. All participants and their legally acceptable guardians or parent(s) provided written informed consent before enrollment. The trial adhered to the principles of the Declaration of Helsinki and to the Good Clinical Practice guidelines of the International Council for Harmonisation for Pharmaceuticals for Human Use.

Adolescents 16–17 years of age were eligible to participate if they were healthy according to medical history, targeted physical examination, and vital sign observations; had normal immune function, no comorbidities potentially associated with an increased risk of severe COVID-19 (including moderate-to-severe asthma, diabetes mellitus, serious heart conditions, and obesity); had no genetic, neurologic, or metabolic disorders; and were polymerase chain reaction–negative for SARS-CoV-2 past or current infection at screening.

The primary objective for the adolescent cohort was to assess the safety and reactogenicity of 1 dose of 2.5×10^10^ vp Ad26.COV2.S. Participants aged 16–17 years were randomized 10:1 to receive 1 dose of 2.5×10^10^ vp Ad26.COV2.S (n=30) or placebo (0.9% saline solution; n=3) using an interactive web response system. Central randomization was implemented in this study by or under the supervision of the sponsor. For the adolescent cohort, manual randomization was utilized; randomization was balanced by using randomly permuted blocks.

Safety evaluations in participants aged 16–17 years were to occur prior to proceeding with planned enrollment of adolescents aged 12–15 years. An independent data monitoring committee evaluated safety and reactogenicity data 7 days post-vaccination. Immunogenicity was higher than in adults vaccinated with Ad26.COV2.S at the 5×10^10^ vp dose level. Reactogenicity was also higher than in adults vaccinated with Ad26.COV2.S at the 5×10^10^ vp dose level but considered acceptable by the independent data monitoring committee, which endorsed escalating the dose to 5×10^10^ vp in participants aged 16–17 years. At the same time, the sponsor temporarily paused enrollment in October 2020 while a safety concern of vaccine-induced thrombotic thrombocytopenia, also known as thrombosis with thrombocytopenia syndrome (a serious adverse event observed in a phase 3 study of adults), was evaluated across all Ad26.COV2.S studies. After the study pause, the sponsor decided to stop further recruitment based on the reactogenicity and immunogenicity data from the 2.5×10^10^ vp dose in the adolescent participants and to further evaluate vaccination of a larger adolescent population in a separate pediatric study (COV3006; ClinicalTrials.gov Identifier: NCT05007080). Enrolled participants continued protocol-defined activities other than vaccination. Participants in the placebo group were allowed to cross over to receive 2.5×10^10^ vp Ad26.COV2.S at 6 months after initial vaccination.

## Vaccine Dosage and Administration

Ad26.COV2.S was administered by intramuscular injection into the deltoid muscle. For the 5×10^10^ vp dose administered to adults, 0.5 mL was withdrawn from 1 vial containing 0.75 mL of 1×10^11^ vp/mL. For the adolescent dose of 2.5×10^10^ vp, 0.75 mL of formulation buffer was added to a vial containing 0.75 mL of 1×10^11^ vp/mL, providing 5×10^10^ vp/mL in a vial with an extractable volume >1 mL; 0.5 mL was withdrawn from this vial. Because the appearance of Ad26.COV2.S was slightly different than placebo, blinding was guaranteed by preparation of the study vaccine or placebo by an unblinded pharmacist or other qualified study-site personnel who masked the syringe with translucent yellow blinding tape. The administration of the vaccine or placebo was performed by a blinded study vaccine administrator. Investigators were blinded to the intervention assignment; blinding could be broken in an emergency situation.

## Safety and Reactogenicity Evaluations

Participants were observed for 60 minutes post-vaccination to monitor for acute reactions and solicited AEs. In addition, for 7 days post-vaccination, participants were asked to record solicited local AEs (vaccination-site pain/tenderness, erythema, and swelling) and solicited systemic AEs (fatigue, headache, nausea, myalgia, and fever [body temperature ≥38°C]) daily. The investigator discussed the recorded information with the participant and legal guardian at the Day 7 safety visit and documented the results on the electronic case report form. Solicited AEs were graded in severity from 1–4 (**Supplementary Tables 2** and **3**). Unsolicited AEs were recorded from the time of vaccination until 28 days post-vaccination and captured on the electronic case report form. All serious AEs and AEs leading to discontinuation from the study were recorded for all participants from the moment of first vaccination until completion of the participant’s last study‑related procedure. Participants with AEs were followed until resolution or until clinically stable.

## Immunogenicity Evaluations

The secondary objective for adolescent participants was to assess humoral immune responses to 1 dose of 2.5×10^10^ vp Ad26.COV2.S. Humoral responses, including binding and neutralizing activity, were measured at Days 1 (baseline), 29, 57, 85, and 169 post-vaccination by a spike protein–specific enzyme-linked immunosorbent assay (S-ELISA) and a wild-type virus-neutralization assay (wtVNA).

Immunogenicity was assessed based on baseline SARS-CoV-2 seropositivity status. A participant was considered SARS-CoV-2 seropositive at baseline if they met either of the following criteria: 1) positive for S-ELISA and/or nucleocapsid enzyme-linked immunosorbent assay (N-ELISA) at Day 1; or 2) polymerase chain reaction SARS-CoV-2 positive at screening.

### *S-ELISA*

The concentration of binding antibodies specific for SARS-CoV-2 prefusion conformation spike protein was determined using the human SARS-CoV-2 pre-spike immunoglobulin G indirect enzyme-linked immunosorbent assay (ELISA). The SARS-CoV-2 antigen used was a stabilized prefusion spike protein ([2P], Δfurin, T4 foldon, His Tag) derived from the first clinical isolate of the Wuhan strain (Wuhan, 2019, whole-genome sequence NC_045512). The S-ELISA (validated with adult serum and cross-checked with pediatric material) was developed for adult and pediatric human serum at Nexelis, a Q^2^ solutions company (Laval, Canada).

Purified SARS-CoV-2 pre-spike antigen was adsorbed to the wells of a microplate and incubated with diluted serum samples, including test samples, standard samples, and quality controls. Unbound sample was washed away, followed by incubation with an enzyme-conjugated antihuman immunoglobulin G and colorimetric detection with 3,3′,5,5′-tetramethylbenzidine. A reference standard on each test plate was used to quantify the antibodies against SARS-CoV-2 pre-spike protein in the sample according to the unit assigned by the standard (ELISA laboratory unit per milliliter [EU/mL]). The ELISA antibody concentrations were calculated from a 4-PL curve generated from 2-fold serial dilution for each sample. In every run, parallelism between sample and the standard was ensured by calculating the interdilutional %CV.

### *wtVNA*

The wild-type virus stocks were derived from the Victoria/1/2020 strain. The wtVNA was performed by adding SARS-CoV-2 wild-type virus to serum samples (working concentration of approximately 100 plaque-forming units/well) and incubating at 37°C for 60–90 minutes. The serum-virus mixture was then transferred onto assay plates and incubated (37°C; 5% CO_2_) for 60–90 minutes with Vero E6 African green monkey kidney cells before the addition of carboxymethyl cellulose overlay medium for 24 hours. Cells were then fixed and stained using SARS-CoV-2 receptor–binding domain spike protein–specific antibodies, and immunoplaques were visualized and counted. Neutralizing titers were calculated as the reciprocal serum dilution corresponding to the 50% neutralization antibody titer for that sample. The wtVNA (validated with adult serum and cross-checked with pediatric material) was developed for adult and pediatric human serum at UK Health Security Agency (Porton, United Kingdom).

*Responder Definitions*

For both immunogenicity assays, the responder definition for post-vaccination samples were: if the baseline sample was negative, a positive result was considered a responder; if the baseline sample was negative, the post-vaccination sample was considered a responder if there was a ≥4-fold increase from baseline.

## Analysis Sets

Any sample taken after a participant met the criteria for a protocol deviation expected to impact immunogenicity outcomes or after the participant experienced any natural SARS-CoV-2 infection was excluded from the per-protocol immunogenicity (PPI) set. Descriptive statistical analyses were performed with SAS analysis software (ie, calculation of geometric mean and CIs, or median and interquartile range Q1–Q3, as appropriate).

# Supplementary Results

## Participant Demographics and Disposition

Baseline demographic information is presented in **Supplementary Table 1**. Forty-four adolescent participants were screened, out of whom 33 were randomized 10:1 to receive 1 dose of Ad26.COV2.S (n=30) or placebo (n=3). Three participants were retrospectively identified in the vaccine group as baseline SARS-CoV-2 seropositive (2 nucleocapsid seropositive; 1 spike seropositive) and were excluded from the PPI set; none of the participants in the placebo group were seropositive at baseline.

## Safety and Reactogenicity

Solicited local AEs were reported by 29 participants in the vaccine group and 1 in the placebo group (**Figure 1A**). The most frequently reported AE was vaccination-site pain (vaccine, 97%; placebo, 33%), the majority of which were grade 1 or 2 in severity. Grade 3 vaccination-site pain occurred in 10% of participants in the vaccine group. Two participants in the vaccine group reported grade 1 vaccination-site swelling. No grade 4 solicited local AEs were reported in either group.

Solicited systemic AEs were reported by 28 participants in the vaccine group and all 3 participants in the placebo group (**Figure 1A**). The most commonly reported events included headache, fatigue, and myalgia, reported by 83%, 70%, and 53% of adolescents in the vaccine group, respectively. Grade 3 events of headache, fatigue, and myalgia occurred in 23%, 10%, and 7% of vaccine recipients, respectively. These grade 3 solicited systemic events lasted fewer than 3 days. Nausea was reported by 27% of those in the vaccine group, with 1 participant reporting a grade 3 event. Fever occurred in 17% of participants in the vaccine group; all events were grade 1 or 2. Grade 1 fatigue was reported by all 3 participants in the placebo group. Headache and myalgia were reported in 1 placebo group participant each; both were grade 1. No grade 4 solicited systemic AEs were reported in either group.

Local and systemic reactogenicity were observed in a higher proportion of adolescents compared with adults or young adults aged 18–25 years who were vaccinated with 5×10^10^ vp Ad26.COV2.S (**Figures 1B** and **1C**). Vaccination-site pain was the most frequently reported solicited local AE (adolescents, 97%; all adults, 50%; younger adults, 73%). Adolescents and younger adults both reported higher rates of any solicited systemic AEs (93% and 97%, respectively) than all adults (61%; **Figures 1B** and **1C**). Proportions of any grade 3 solicited systemic AE were higher in adolescents (23%) than adults (3%) or young adults aged 18–25 years (12%). Fatigue, headache, and myalgia were the most common solicited systemic AEs in all age groups, with generally higher rates of grade 3 events in adolescents. Although rare, grade 3 fever was reported more frequently among all adults and younger adults (1% and 3%, respectively) than adolescents (0%).

Unsolicited AEs were reported by 12 (40%) adolescents in the vaccine group, with the most common event being chills (10%); 2 (67%) adolescents in the placebo group reported multiple unsolicited AEs (see **Supplementary Table 4**). The majority of unsolicited AEs were grade 1 or 2 in severity. One participant experienced a grade 3 event of chills that was considered related to vaccination. No serious AEs, thrombotic events, or AEs leading to death were reported in adolescents.

## Immunogenicity

Thirty participants who were seronegative at baseline (vaccine, n=27; placebo, n=3) were included in the PPI set. In the vaccine group, baseline geometric mean concentrations (GMCs; EU/mL) of spike protein–specific binding antibodies were below the lower limit of quantification (LLOQ). By Day 29, all seronegative adolescents had humoral immune responses (**Supplementary Figure 1A**). The GMC (95% CI) was 682 (506–920) by Day 29; GMCs slightly increased through Day 57 (770 [593–1000]), stabilized by Day 85 (773 [547–1093]), and were maintained up to Day 169 (796 [449–1411]). Response rates were maintained at 100% from Day 29 through Day 169. No binding antibodies were detected in the placebo group at any time point.

The 3 adolescents who were seropositive for SARS-CoV-2 at baseline and received Ad26.COV2.S had robust responses from baseline (GMC 61 [<LLOQ–563]) to Day 29 (4410 [101–>ULOQ]), reflecting a mean 57.4-fold increase in spike protein–binding antibody levels compared with a mean 13.4-fold increase in participants who were seronegative at baseline. Binding antibody levels declined in the seropositive participants through Day 169 (918 [<LLOQ–>ULOQ]), with levels 10.6-fold higher than baseline.

GMCs of spike protein–binding antibodies in all adolescents from the vaccine group (2.5×10^10^ vp Ad26.COV2.S) were higher than in adults aged 18–55 and ≥65 years who received a single dose of 5×10^10^ vp Ad26.COV2.S (**Supplementary Figure 1B, right panel**). At Day 29, adult GMCs increased from <LLOQ at baseline to 360 (276–471) and continued to increase by Day 57 (496 [381–647]). By Day 85, adult GMCs were maintained at 476 (357–634), with a slight decline by Day 169 (337 [242–468]). Response rates were slightly higher in adolescents (100% at all time points) than in adults 18–55 and ≥65 years (range, 91%–98%). Although the 95% CIs largely overlapped, GMCs in vaccinated adolescents were lower than those in young adults aged 18–25 years (5×10^10^ vp dose level) at Day 29 (774 [528–1134]), Day 57 (1057 [598–1871]), and Day 85 (1064 [609–1861], **Supplementary Figure 1B, left panel**); GMCs were comparable in adolescents compared with young adults at Day 169 (796 [449–1411] vs 779 [376–1617], respectively). Young adults who received the 5×10^10^ vp dose level also had response rates of 100% at Day 29 that were maintained through Day 169.

At baseline, neutralizing antibodies assessed by wtVNA were <LLOQ for both the vaccine and placebo groups. By Day 29, geometric mean titers (GMTs [95% CI]) increased to 305 (245–378) in vaccinated adolescents (**Supplementary Figure 2A**). GMTs in vaccinated adolescents remained stable through Day 169 (Day 57: 323 [262–399]; Day 85: 306 [227–413]; Day 169: 342 [219–532]). Response rates reached 100% at Day 29 and were maintained through Day 169. Neutralizing antibody responses were not elicited in the placebo group at any time point.

Among the 3 adolescents who were baseline seropositive for SARS-CoV-2, Ad26.COV2.S vaccination appeared to boost pre-existing humoral immune responses, with a mean 15.4-fold increase in neutralizing antibody titers from baseline (GMT 65 [<LLOQ–2134]) to Day 29 (1589 [<LLOQ–>ULOQ]) compared with a mean 5.2-fold increase in baseline seronegative participants. Neutralizing antibody titers declined in the seropositive participants through Day 169, with titers 3.5-fold higher than baseline (479 [<LLOQ–>ULOQ]).

GMTs in adolescents receiving 2.5×10^10^ vp Ad26.COV2.S were higher than in adults 18–55 and ≥65 years receiving the 5×10^10^ vp dose level (**Supplementary Figure 2B, right panel**). At Days 29 and 57, adult GMTs increased from <LLOQ at baseline to 253 (207–308) and 259 (173–386), respectively. By Day 85, adult GMTs slightly decreased to 209 (140–311), with further decline at Day 169 (154 [95–250]). Response rates were 100% in both adolescents and adults at Day 29; response rates were higher in adolescents (100%) than adults (range, 75%–90%) at the remaining time points. From Day 29 through Day 169, neutralizing antibodies in vaccinated adolescents were lower than in young adults aged 18–25 years receiving 5×10^10^ vp Ad26.COV2.S (**Supplementary Figure 2B, left panel**). In young adults, GMTs increased from <LLOQ at baseline to 376 (277–509) at Day 29, peaked at Day 57 (561 [219–1435]), and slightly declined through Day 85 (507 [216–1191]) and Day 169 (400 [135–1188]). Response rates reached 100% by Day 29 and were maintained through Day 169. Differences between adolescents and young adults aged 18–25 should be interpreted with caution as the sample size of young adults was small (n=6 at Days 29 and 57; n=7 at Days 85 and 169).

**Supplementary Table 1.** Adolescent Participant Baseline Demographic Characteristics

| **Characteristic** | **Ad26.COV2.S (n=30)** | **Placebo (n=3)** | **Full analysis set  (N=33)** |
| --- | --- | --- | --- |
| Age, years |  |  |  |
| Median (range) | 16.3 (16–17) | 16.3 (16–17) | 16.0 (16–17) |
| Sex, n (%) |  |  |  |
| Female | 20 (67) | 2 (67) | 22 (67) |
| Male | 10 (33) | 1 (33) | 11 (33) |
| Race, n (%) |  |  |  |
| Black or African American | 1 (3) | 0 | 1 (3) |
| White | 29 (97) | 3 (100) | 32 (97) |
| Hispanic ethnic group, n (%) |  |  |  |
| Not Hispanic or Latino | 29 (97) | 2 (67) | 31 (94) |
| Not reported | 1 (3) | 1 (33) | 2 (6) |
| Body mass index, kg/m^2^ |  |  |  |
| Median (range) | 20.9 (17.0–28.2) | 18.4 (18.2–22.9) | 20.8 (17.0–28.2) |
| Country, n (%) |  |  |  |
| Spain | 19 (63) | 0 | 19 (58) |
| United Kingdom | 11 (37) | 3 (100) | 14 (42) |

**Supplementary Table 2.** Grading Scale for Local Solicited Adverse Events^a^

| **Local reaction** | **Mild  (grade 1)** | **Moderate  (grade 2)** | **Severe  (grade 3)** | **Potentially life-threatening  (grade 4)** |
| --- | --- | --- | --- | --- |
| Pain/tenderness^b^ | - Aware of symptoms but easily tolerated - Does not interfere with activity - Discomfort only to touch | - Notable symptoms - Requires modification in activity or use of medications - Discomfort with movement | - Incapacitating symptoms - Inability to do work, school, or usual activities - Use of narcotic pain reliever | - Hospitalization - Pain/tenderness causing inability to perform basic self-care functions |
| Erythema^b^ | - 25–50 mm | - 51–100 mm | - >100 mm | - Hospitalization - Necrosis or exfoliative dermatitis |
| Swelling^b^ | - 25–50 mm | - 51–100 mm | - >100 mm | - Hospitalization - Necrosis |

^a^Grading scale adapted from US Food and Drug Administration, Center for Biologics Evaluation and Research. September 2007. Guidance for industry. Toxicity grading scale for healthy adult and adolescent volunteers enrolled in preventive vaccine clinical trials. Accessed September 28, 2022. https://www.fda.gov/downloads/BiologicsBloodVaccines/GuidanceComplianceRegulatoryInformation/Guidances/Vaccines/ucm091977.pdf.

^b^Revised by the sponsor.

**Supplementary Table 3.** Grading Scale for Systemic Solicited Adverse Events^a^

| **Systemic** | **Mild  (grade 1)** | **Moderate  (grade 2)** | **Severe  (grade 3)** | **Potentially life-threatening  (grade 4)** |
| --- | --- | --- | --- | --- |
| Nausea/vomiting | - No interference with activity or 1–2 episodes/24 hours | - Some interference with activity or >2 episodes/24 hours | - Prevents daily activity, requires outpatient IV hydration | - Hospitalization for hypotensive shock |
| Headache | - No interference with activity | - Repeated use of nonnarcotic pain reliever for >24 hours or some interference with activity | - Significant; any use of narcotic pain reliever or prevents daily activity | - Hospitalization |
| Fatigue | - No interference with activity | - Some interference with activity | - Significant; prevents daily activity | - Hospitalization |
| Myalgia | - No interference with activity | - Some interference with activity | - Significant; prevents daily activity | - Hospitalization |
| Fever, ℃ | - 38.0–38.4 | - 38.5–38.9 | - 39.0–40 | - >40 |

^a^Grading scale adapted from US Food and Drug Administration, Center for Biologics Evaluation and Research. September 2007. Guidance for industry. Toxicity grading scale for healthy adult and adolescent volunteers enrolled in preventive vaccine clinical trials. Accessed September 28, 2022. https://www.fda.gov/downloads/BiologicsBloodVaccines/GuidanceComplianceRegulatoryInformation/Guidances/Vaccines/ucm091977.pdf.

**Supplementary Table 4.** Unsolicited Adverse Events in Adolescent Participants Who Received 2.5×10^10^ vp Ad26.COV2.S Vaccine or Placebo

| **Full analysis set, n (%)** | **Ad26.COV2.S**  **n=30** | **Placebo**  **n=3** |
| --- | --- | --- |
| Participants with ≥1 adverse event | 12 (40) | 2 (67) |
| Eye disorders | 1 (3) | 1 (33) |
| Episcleritis | 1 (3) | 0 |
| Eye pain | 0 | 1 (33) |
| Gastrointestinal disorders | 1 (3) | 1 (33) |
| Abdominal pain | 0 | 1 (33) |
| Toothache | 1 (3) | 0 |
| General disorders and administration-site conditions | 3 (10) | 1 (33) |
| Chills^a^ | 3 (10) | 0 |
| Fatigue | 0 | 1 (33) |
| Vaccination-site pain | 1 (3) | 0 |
| Infections and infestations | 2 (7) | 0 |
| Tonsillitis | 1 (3) | 0 |
| Upper respiratory tract infection bacterial | 1 (3) | 0 |
| Injury, poisoning, and procedural complications | 2 (7) | 0 |
| Chemical burn | 1 (3) | 0 |
| Joint injury | 1 (3) | 0 |
| Musculoskeletal and connective tissue disorders | 1 (3) | 0 |
| Arthralgia | 1 (3) | 0 |
| Nervous system disorders | 3 (10) | 0 |
| Dizziness | 1 (3) | 0 |
| Presyncope | 1 (3) | 0 |
| Syncope | 1 (3) | 0 |
| Reproductive system and breast disorders | 1 (3) | 1 (33) |
| Dysmenorrhea | 1 (3) | 0 |
| Premenstrual pain | 0 | 1 (33) |

vp, viral particles.

^a^One participant in the vaccine group experienced a grade 3 event considered to be related to vaccination.

**Supplementary Figure 1.** SARS-CoV-2 spike protein–binding antibody levels elicited by (A) 1 dose of 2.5×10^10^ vp Ad26.COV2.S or placebo in seronegative adolescents 16–17 years of age compared to (B) adults from the same study who received 1 dose of 5×10^10^ vp Ad26.COV2.S who were 18–25 years of age and 18–55 and ≥65 years of age. GMC with 95% CI is shown. Arrows in (B) indicate vaccination with 5×10^10^ vp Ad26.COV2.S at Day 1, placebo at Day 57, and 1.25×10^10^ vp Ad26.COV2.S at Day 169. EU/mL, ELISA laboratory unit per milliliter; GMC, geometric mean concentration; LLOQ, lower limit of quantification; ULOQ, upper limit of quantification; vp, viral particles.


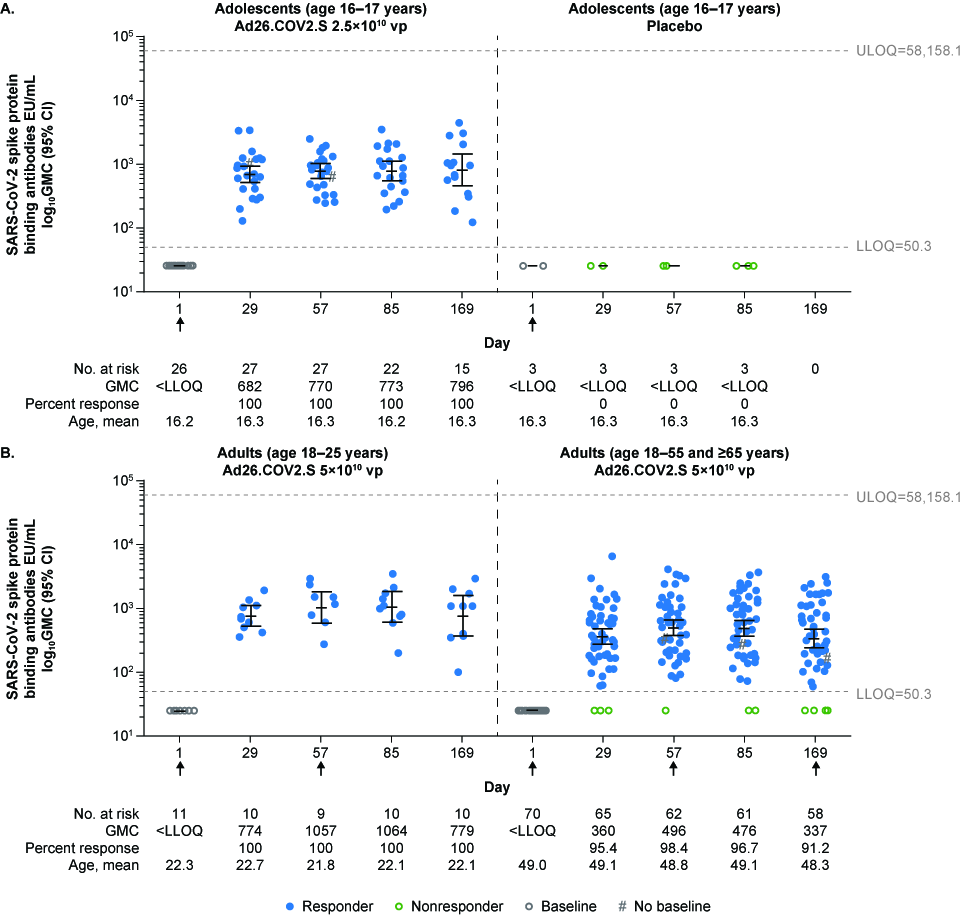


**Supplementary Figure 2.** Neutralizing wtVNA titers elicited by (A) 1 dose of 2.5×10^10^ vp Ad26.COV2.S or placebo in adolescents 16–17 years of age compared to (B) adults from the same study who received 1 dose of 5×10^10^ vp Ad26.COV2.S who were 18–25 years of age and 18–55 and ≥65 years of age. GMT with 95% CI is shown. Arrows in (B) indicate vaccination with 5×10^10^ vp Ad26.COV2.S at Day 1, placebo at Day 57, and 1.25×10^10^ vp Ad26.COV2.S at Day 169. GMT, geometric mean titer; IC_50_, half-maximal inhibitory concentration; LLOQ, lower limit of quantification; ULOQ, upper limit of quantification; wtVNA, wild-type virus-neutralization assay; vp, viral particles.


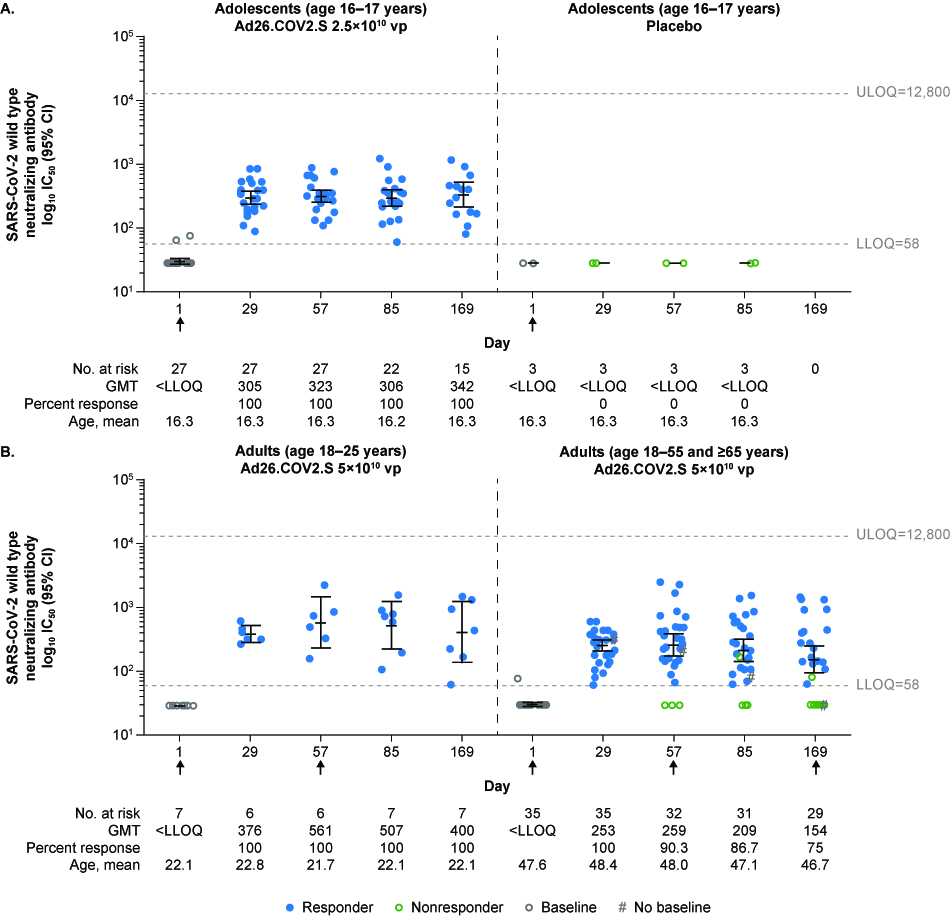

Supplement: piae098_suppl_Supplementary_Materials [file piae098_suppl_supplementary_materials.docx]
